# Physical Simulation and Numerical Simulation of Flash Butt Welding for Innovative Dual Phase Steel DP590: A Comparative Study

**DOI:** 10.3390/ma16093513

**Published:** 2023-05-03

**Authors:** Jingwen Song, Lisong Zhu, Jun Wang, Yao Lu, Cheng Ma, Jian Han, Zhengyi Jiang

**Affiliations:** 1 School of Materials Science and Engineering, Tianjin University of Technology, Tianjin 300384, China; 2 School of Mechanical, Materials, Mechatronic and Biomedical Engineering, University of Wollongong, Wollongong, NSW 2522, Australia; 3 Welding and Additive Manufacturing Centre, Cranfield University, Cranfield, MK43 0AL, UK; 4 Technology Research Institute, HBIS Group, Shijiazhuang 050023, China

**Keywords:** flash butt welding, dual-phase steel, thermal-mechanical simulation, numerical simulation, heat affected zone, softening behaviour

## Abstract

In this study, the microstructure and performance of newly designed dual-phase steel (DP590) after joining by flash butt welding (FBW) for vehicle wheel rims was analysed and compared by two simulations, i.e., physical simulation and numerical simulation, due to the high acceptance of these two methodologies. Physical simulation is regarded as a thermal–mechanical solution conducted by the Gleeble 3500 simulator and which can distribute the heat-affected zone (HAZ) of the obtained weld joint into four typical HAZs. These are coarse-grained HAZ, fine-grained HAZ, inter-critical HAZ and sub-critical HAZ. A combination of ferrite and tempered martensite leads to the softening behaviour at the sub-critical HAZ of DP590, which is verified to be the weakest area, and influences the final performance due to ~9% reduction of hardness and tensile strength. The numerical simulation, relying on finite element method (FEM) analysis, can distinguish the temperature distribution, which helps us to understand the relationship between the temperature distribution and real microstructure/performance. Based on this study, the combination of physical and numerical simulations can be used to optimise the flash butt welding parameters (flash and butt processes) from the points of temperature distribution (varied areas), microstructure and performance, which are guidelines for the investigation of flash butt welding for innovative materials.

## 1. Introduction

Developing lightweight materials, saving energy, and reducing carbon emissions and other pollutants are inevitable trends in the automotive industry [1,2,3]. Besides these drives, safety is an eternal topic regarding vehicles. In general, wheels can easily suffer from extreme damage during driving, and the wheel rim is the critical part that directly determines the safety of vehicles. Therefore, when selecting appropriate materials and in subsequent processing, reliable performance is of crucial importance for wheel rims.

In recent years, dual-phase (DP) steels have been considered for application in manufacturing wheels due to their flexible control of performance and controllable costs [4]. Compared to high-strength low-alloyed (HSLA) steel, DP steels rely on lean alloying design, aiming at strength improvement through phase control [5]. In this case, the performance of the DP steels can be adjusted and optimised by phase type and fraction. Therefore, the replacement of HSLA steel with DP steel can reduce expected costs, and, meanwhile, major performance can be remained.

As an important classification of resistance welding technology, flash butt welding (FBW) has been extensively applied in industry, such as in building railway tracks and oil/gas pipelines. For instance, in railway tracks, the FBW is applied for joining tracks with high efficiency and quality [6,7,8,9]. During flash butt welding, the contact surfaces of a pair of workpieces are heated with a rapid heating rate until they melt and join by the existing forces. This is a flashing process. After that, the workpieces are pressed by the axial displacement of the electrodes, in order to extrude the impurities out from the melted surfaces. This is the upsetting process [10]. In the FBW process, the upsetting process plays a role in forging, which can provide good mechanical performance for the joint. Therefore, FBW is an efficient welding process, and is regarded as a combination of high heat input and extra forging. Furthermore, the FBW process does not need any consumables, which can save money in mass production and processing [11,12,13].

FBW is widely applied in manufacturing wheel rims due to its high heat efficiency and excellent formability [7,8]. Xi et al. [12] reported processing parameters of FBW, which were adjusted and optimised to improve the mechanical properties of RS590CL HSLA steel, and the suitable combination of flash allowance, upset allowance and speed was beneficial for the improvement of joint performance. However, some problems will still be generated during FBW of steels. For instance, when HSLA 590CL is processed by FBW, the softening behaviour results in ductile fractures in the heat-affected zone (HAZ), and the coarsened grains lead to brittle fractures in the weld zone (WZ) [14,15]. Another case is an ultra-fine grain steel (a special Q235C steel with the average grain size of ~7 μm). In one study, the softening behaviour caused by FBW still appeared. However, there was only a localised softening area inside the weld joint, and this did not influence the properties of the whole joint [16]. The procedure parameters used in FBW processing can influence the performance of the products. Here, the DP steel is sensitive to temperature. The heat input from welding can cause changes to the microstructure, particularly the martensite islands. DP steel generally has a problem of softening behaviour at the HAZ when welding. In this case, studying the softening is very important. According to these softening mechanisms, strategies can be proposed to modify the mechanical performances of the FBW-ed products [17].

In this study, both physical simulation and numerical simulation were applied to understand the relationship between temperature distribution and microstructure/performance to evaluate the flash butt welding parameters. The numerical simulation was applied to study the FBW process first [18]. In general, the electrical–thermal bidirectional coupling method is used in the finite element method (FEM) simulation of FBW. However, numerical simulations regarding the FBW process of wheel rims have not been investigated in depth. Further, in order to control the production cost, the Gleeble simulator was relied on to simulate the FBW process using the thermal cycles captured from real welding [3,19].

In this study, systematic FBW simulations, including physical simulations and numerical simulations, plus characterisation of the microstructure and performance regarding a novel DP steel were executed, aiming at precisely evaluating the safety of newly developed wheel steels. The methodologies proposed in this study can evaluate the feasibility of FBW-ed DP steels with high efficiency and low cost before actual production.

## 2. Experiments and Simulations

### 2.1. Experimental Procedure

**Figure 1:**
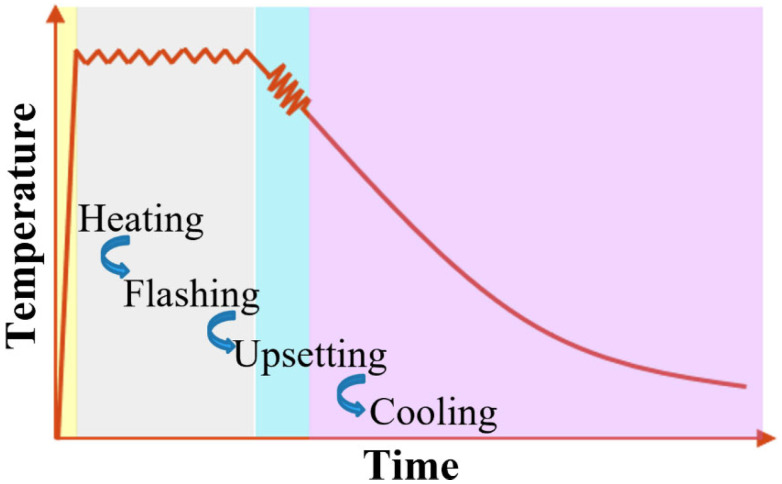
Thermal history during the flash butt welding process.

**Figure 2:**
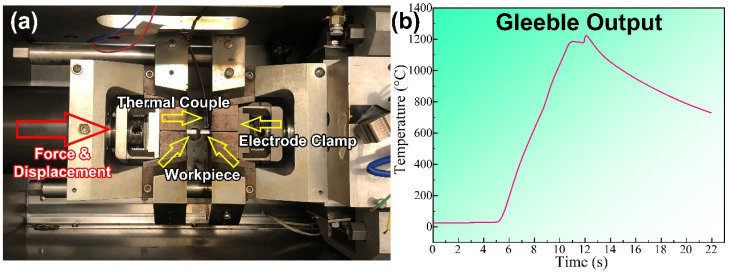
(**a**) Gleeble 3500 system at UOW. (**b**) Temperature–time curve captured in a FBW Gleeble simulation.

Figure 1 shows the designed thermal history of the sample during the FBW process. The workpieces were firstly heated to the peak temperature, and then flashing was conducted under an isothermal process to maintain the melting. After flashing, the upsetting was started. During the upsetting, the temperature of the workpieces was kept at a fixed value, which was maintained for the upsetting duration in the speed set. Newly developed hot-rolled DP590 wheel steel plates with dimensions of 42 mm (length) × 9.1 mm (width) × 4.9 mm (thickness) were designed to complete the physical simulation using a Gleeble 3500 simulator (Dynamic Systems Inc., Austin, TX, USA) at the University of Wollongong (UOW), as shown in Figure 2a. The chemical composition of DP590 steel (HBIS Group, Shijiazhuang, China) is listed in Table 1. In the simulations, the welding cycles (peak temperature: 1150 °C) were designed and carried out based on the thermal cycles in real welding, which occurred 10 mm away from the contact surface; the thermal curve captured from the thermocouples is shown in Figure 2b. The Gleeble simulation test was repeated three times and the output results of the other two tests are shown in Figure S1 (Supplementary Materials). The processing parameters for the physical simulation are as follows: 6 mm flash allowance; 5 mm/s flash speed; 6.5 mm upset allowance; 20 mm/s upset speed. The continuous cooling curves were detected using a dilatometer under different cooling rates.

The CALPHAD software “Thermo-Calc” (Thermo-Calc Software, Stockholm, Sweden) was used to evaluate the phase transition temperatures of the DP590 steel. The FE analysis for the thermal–mechanical processing during the FBW was conducted using Abaqus (Dassault Systèmes Simulia Corp., Velizy Villacoublay, France). The fractions of different phases in the DP590 steel were evaluated using ImageJ (National Institutes of Health, Bethesda, ML, USA). A Nikon ECLIPSE LV100NDA optical microscope (OM; Nikon Metrology NV, Leuven, Belgium), and a JSM-7001F scanning electron microscope (SEM; JEOL Ltd., Tokyo, Japan) equipped with energy dispersive spectroscopy (EDS) was used to observe the microstructures and to measure the elemental distribution. A MATSUZAWA Via-F micro-hardness tester (Matsuzawa Co., Ltd., Akita Pref, Japan) was used to test the hardness at 500 g for 10 s. An Instron tensile tester (Illinois Tool Works Inc., Glenview, IL, USA) was used to test the strengths of the samples. To precisely detect the strain of the sample under the tensile test, the length variation of the sample was observed and measured using a camera.

### 2.2. FEM Simulation

**Figure 3:**
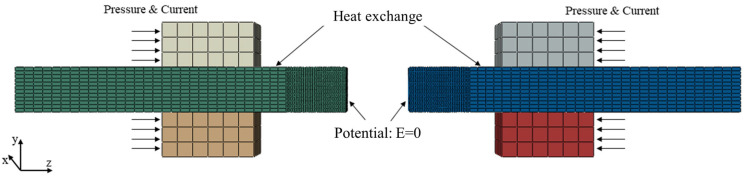
Geometry model of FEM simulation.

The FEM simulations of the FBW process were conducted using Abaqus. Figure 3 shows the model of the FEM simulation. The size of the workpiece in the FEM simulation was the same as in the Gleeble simulation, i.e., 42 mm (length) × 9.1 mm (width) × 4.9 mm (thickness). The processing parameters for FEM simulation were as follows: 6 mm flash allowance; 5 mm/s flash speed; 6.5 mm upset allowance; 20 mm/s upset speed, which are the same as the Gleeble simulation. In this numerical simulation, the expression of high-temperature rheological properties of DP590 steel was based on the Arrhenius constitutive model developed by Liu et al. [20]. Here, the activation energy was 508.29 kJ·mol^−1^. The physical parameters used in this simulation are given in Table 2. The thermoelectric property of the contact surface and the thermal conductivity of the electrode and workpiece are listed in Table S1 (Supplementary Materials).

Firstly, the electric field distribution was simulated. According to the theory of electromagnetic fields, when the current flows through the conductor section, the voltage distribution can be described by the Laplace equation [21,22]:

(1) $$\frac{\partial }{\partial x}\left(\sigma \frac{\partial U}{\partial x}\right)+\frac{\partial }{\partial y}\left(\sigma \frac{\partial U}{\partial y}\right)+\frac{\partial }{\partial z}\left(\sigma \frac{\partial U}{\partial z}\right)=0$$

where *σ* is conductivity (Ω^−1^·m^−1^) and *U* is electric potential (V).

The electrode voltage applied in the FEM simulation was set to 220 V, and the potential between the contact surfaces was set to zero. In the process of flash butt welding, the resistance of the welding zone consists of the contact resistance and the body resistance of the workpiece itself. The contact resistance is much larger than the body resistance, which plays a leading role in the heating of the workpiece. Several layers of elements endowed with contact resistance were divided from the welding surfaces, and the thickness of each layer was 1 mm. The contact resistance value can be obtained from the empirical formula [21,22]:

(2) R=9500KS23Vf13J×10−6

where *R* is the contact resistance (Ω), *K* is the property coefficient of steel, *S* is the cross-sectional area of the weldment (mm^2^), *V_f_* is flash speed (mm/s), and *J* is current density (A/mm^2^). Generally, the value of the property coefficient (*K*) of steel is set to 1 [21].

The electrical–thermal couple module was used to heat the workpiece. The birth–death element method is usually used to simulate the flash procedure (burning loss of the material) in FEM simulations, i.e., killing the elements which are burned in a real process. However, the calculated temperature field cannot be assigned to the thermal–mechanical coupled model after the cell is killed. Hence, the flash procedure was controlled by changing the electrical conductivity in this study. The electrical conductivity of the burned elements was set to 0.01 (Ω^−1^·m^−1^) to avoid reheating. To control the flash speed, the element was heated to a set welding time and then “killed”. The flash allowance was controlled by the number of element layers killed.

FBW is a welding method that relies on internal heat source heating. For the transient heat transfer problem heated by an internal heat source, the heat conduction differential equation is as follow [21,22]:

(3) $$\rho c\frac{\partial T}{\partial t}=\frac{\partial }{\partial x}\left(\lambda_{x}\frac{\partial T}{\partial x}\right)+\frac{\partial }{\partial y}\left(\lambda_{y}\frac{\partial T}{\partial y}\right)+\frac{\partial }{\partial z}\left(\lambda_{z}\frac{\partial T}{\partial z}\right)+\;Q_{v}$$

where ρ is the density (Kg/m^3^), *c* is the specific heat (J/Kg·°C), $\lambda_{x},\lambda_{y},\lambda_{z}$ are thermal conductivity in different directions (J/Kg·°C), and $Q_{v}$ is joule heat (J).

The load and boundary conditions are listed in Table 3. The load was electrode voltage (220 V) and the given boundary condition was zero potential on the contact surface (Figure 3). The heat transfer through the contact surface between the electrode and workpiece was set to be the thermal conduction mode. The heat exchange coefficient between the workpiece surface and air was assumed to be constant at 3800 W/m^2^∙°C. The initial temperature of the whole model was the ambient temperature (20 °C). The step time of finite element analysis was controlled by the thickness of the killed layer and the flash speed. The temperature after killing one layer of elements was set as the initial condition for the temperature calculation of the next layer of elements, and this process was continuously cycled until the flash process was finished.

**Table 2:** Physical parameters of DP590 wheel steel [23].

DP590								
Temperature (°C)	25	100	200	300	400	-	-	-
Thermal conductivity (W/(m∙k))	65.3	54.9	45.2	36.2	28.5	-	-	-
Temperature (°C)	100	200	300	400	500	600	700	800
Coefficient of thermal expansion (×10^−6^K^−1^)	12.76	13.66	14.27	15.17	15.52	15.64	15.41	12.73
Temperature (°C)	25	50	100	150	200	300	400	450
Specific heat capacity (J/kg∙°C)	460	468	485	502	519	552	586	602
Temperature (°C)	20	25	100	200	300	400	500	600
Electrical resistivity (×10^−6^Ω∙m)	0.284	0.301	0.335	0.402	0.478	0.564	0.666	0.806

## 3. Results

### 3.1. Microstructure

**Figure 4:**
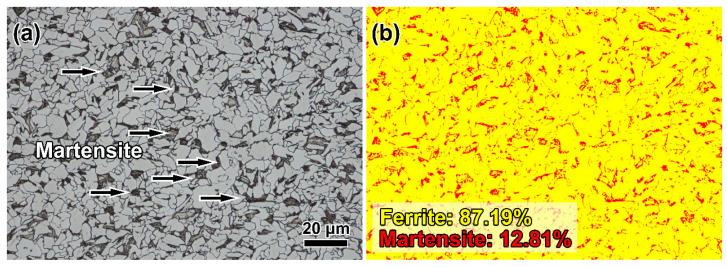
(**a**) Microstructure of the DP590 wheel steel; (**b**) fraction of martensite and ferrite.

**Figure 5:**
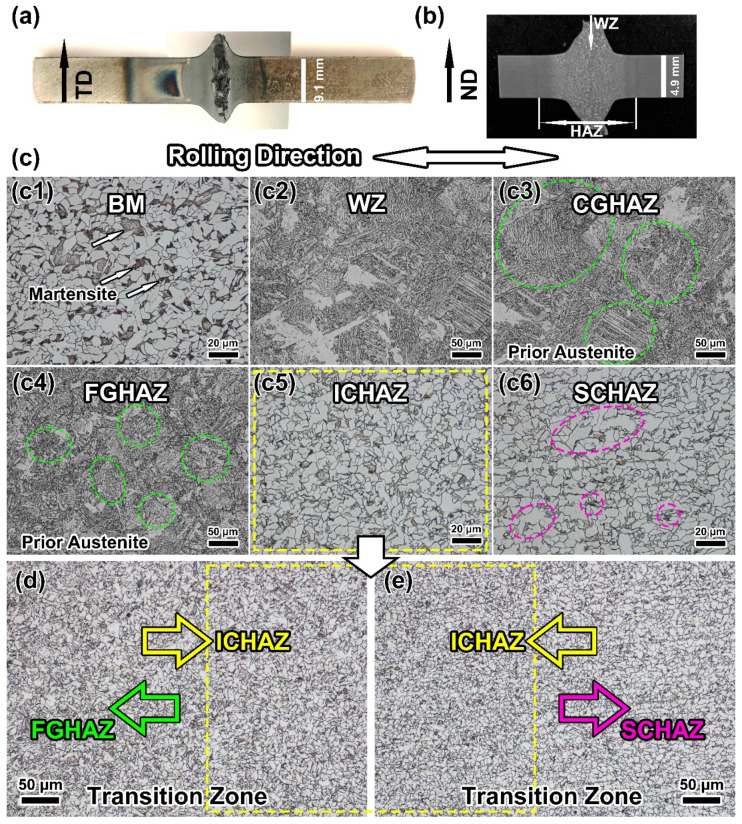
(**a**) Morphology of FBW-ed sample; (**b**) macrostructure of weld joint; (**c**) microstructure of the main heat-affected zones; (**d**) microstructure of the transition zone between FGHAZ and ICHAZ; (**e**) microstructure of the transition zone between ICHAZ and SCHAZ.

The optical microstructure of the DP590 wheel steel base metal (BM) is displayed in Figure 4a. It can be observed that the hot-rolled DP590 steel consists of martensite (M) and polygonal ferrite (PF). As shown in Figure 4b, the fractions of M and PF were evaluated to be 12.81% and 87.19%, respectively. The WZ exhibits typical coarse grains and a Widmanstatten structure. From Figure 5, it is seen that for the DP steel, the HAZ can be divided into four main zones based on the peak temperatures of each zone. These are the coarse-grained heat-affected zone (CGHAZ), the fine-grained heat-affected zone (FGHAZ), the intercritical heat-affected zone (ICHAZ), and the subcritical heat-affected zone (SCHAZ). The positions of the different HAZs are indicated in Figure S2 (Supplementary Materials). The CGHAZ and FGHAZ exhibit apparent lamellar martensite structures and austenite grain boundaries. However, the grains of prior austenite of the CGHAZ are larger than those of the FGHAZ due to higher peak temperatures. A majority of the grains in the CGHAZ were larger than 100 μm. Meanwhile, the grains in the FGHAZ were generally smaller than 50 μm.

The fine ferrite was the major phase in the ICHAZ, together with some remaining tempered martensite. The microstructure of the SCHAZ was very similar to the BM. However, the fraction of martensite in the SCHAZ was significantly lower than that in the BM. Meanwhile, the ferrite grain size in this zone was larger than the BM.

### 3.2. Mechanical Properties

**Figure 6:**
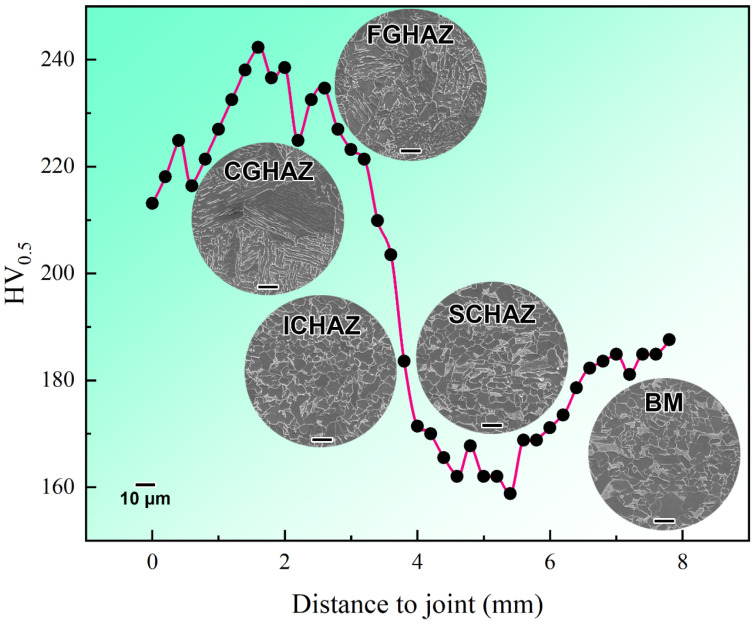
Microhardness of different zones.

The zone of hardness is shown in Figure S2 (Supplementary Materials). The number of tested points in the CGHAZ, FGHAZ, ICHAZ and SCHAZ were 11, 6, 5, and 11, respectively. As shown in Figure 6, the average microhardness values of the different zones were: CGHAZ (232.7 HV_0.5_) > FGHAZ (225.8 HV_0.5_) > ICHAZ (187.7 HV_0.5_) > BM (184.2 HV_0.5_) > SCHAZ (167.2 HV_0.5_). There was a significant decline in the microhardness of the ICHAZ and SCHAZ compared with the CGHAZ and FGHAZ. In terms of the softening behaviour, the hardness of the SCHAZ decreased by 9.2% compared with BM, and the width was 1099.4 μm. The SCHAZ of the DP590 steel was defined to be the abnormal softening area, which needs to match the application requirement. As shown in Figure 5 and Figure 6, CGHAZ and FGHAZ had lamellar martensite structures, which can provide a high hardness. Compared with the CGHAZ and FGHAZ, there was an evident decrease in the hardness in the ICHAZ, which mainly had fine ferrite grains together with some remaining tempering martensite structures. The microstructure of the SCHAZ was very similar to the BM. However, the martensite in the SCHAZ exhibited a characteristic tempering, which is the reason why softening occurred in the SCHAZ.

**Figure 7:**
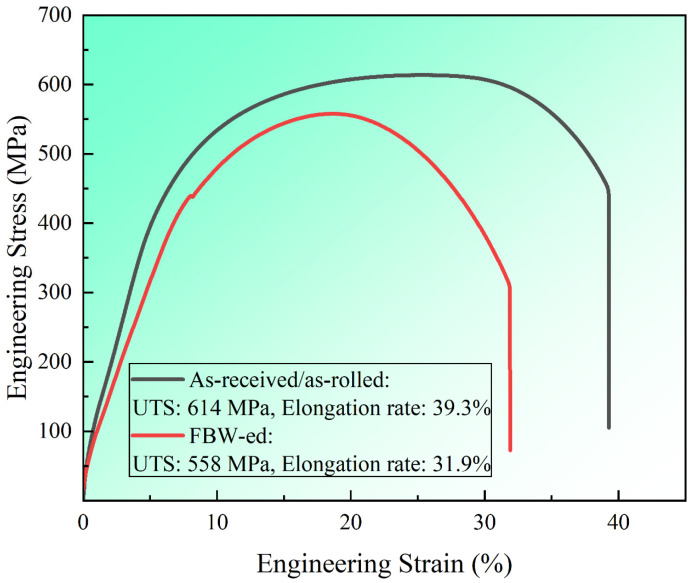
Engineering stress–strain curves of as-received and as-FBW-ed DP590 steel.

Figure 7 shows that the ultimate tensile strength (UTS) of the rolled DP590 steel was ~614 MPa, and the tensile strain was ~39.9%. However, after a flash butt welding process, the UTS (558 MPa) and tensile strain (31.9%) of the FBW-ed sample decreased by 9.1% and 18.8%, respectively. This phenomenon can be attributed to the phase transitions in HAZs.

### 3.3. Thermal Behaviour

**Figure 8:**
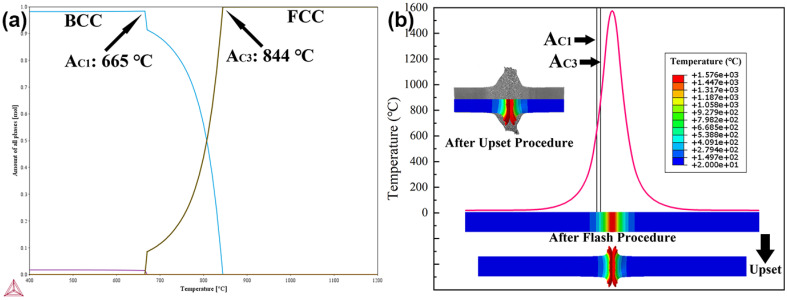
(**a**) Calculated phase diagram of DP590 wheel steel; (**b**) numerical–simulative temperature field of the workpiece.

**Figure 9:**
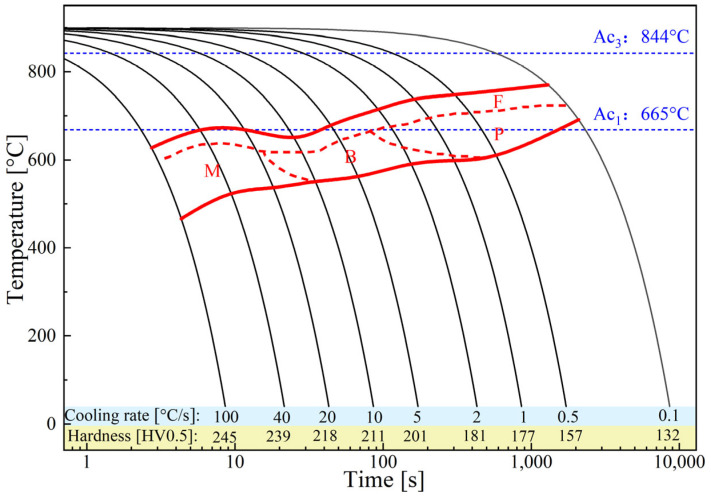
Continuous cooling temperature (CCT) curve of DP590 steel.

The calculated phase diagram of the DP590 steel in Figure 8a shows that the A_C1_ and A_C3_ are 665 °C and 844 °C, respectively. The complete calculated phase diagram is provided in Figure S3 (Supplementary Materials). Figure 8b displays the FEM simulation result, indicating the temperature distribution during FBW. The temperature distributions on the sample length cross section are shown in Figure S4. The temperature gradually decreased from the welding zone to the BM. On the cross section of the sample, the temperature distribution was uniform, and this is an ideal situation. The complete phase transformation led to clear prior austenite grains. The FEM result shows that the temperature of the workpiece displays a decreasing trend from the HAZ to the BM. The temperature of the ICHAZ is lower than the A_C3_. Therefore, incomplete phase transformation occurs at this zone during FBW, and the prior austenite cannot be recognised. Furthermore, the output temperature curve and continuous cooling temperature (CCT) curve (Figure 9) indicate that martensitic transformation occurred in the FGHAZ and CGHAZ during the cooling process. Thus, the massive martensite provides a higher hardness for the CGHAZ and FGHAZ compared with other zones. The peak temperature of the ICHAZ is higher than A_C1_, which means that the phase transformation can still occur in the ICHAZ. As shown in Figure 5(c5),d,e, most of the previous island-shaped martensite structures disappeared in the ICHAZ. In addition, the grains in the ICHAZ are finer than that of the BM and SCHAZ.

**Figure 10:**
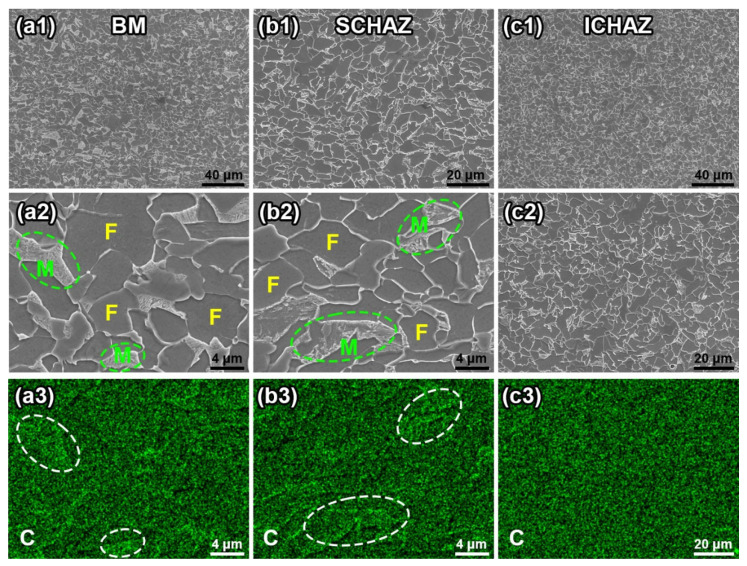
SEM and EDS results of different zones: (**a1**–**a3**) BM; (**b1**–**b3**) SCHAZ; (**c1**–**c3**) ICHAZ.

Figure 10 displays the SEM images of the FBW-ed DP590 steel and distributions of carbon (C). It is obvious that the content of C in martensite is higher than ferrite in the BM. As shown in Figure 5 and Figure 10, the morphology of martensite of the SCHAZ after being tempered is different to that of the BM. It can be observed that the size of the martensite zone of the SCHAZ was smaller than that of the BM. Furthermore, the metallography in Figure 5 shows that the colour of the martensite of the SCHAZ is lighter than the BM. Generally, the phase with a high content of carbon, such as martensite, will display a darker colour under the observation of an optical microscope. With SEM analysis, the phase with a high carbon content will show a brighter colour. However, the carbon in the martensite island is sensitive to temperature. Heating caused by welding makes the diffusion of carbon more active. This phenomenon reduced the carbon content of the martensite island. Figure 10(a3–c3) indicate the differences in C distribution in different zones. Compared with the BM, the carbon content of the martensite island (such as circled zone) in SCHAZ obviously decreased. When it comes to ICHAZ, the carbon element was nearly uniformly distributed. The EDS result in Figure 10 coincides with the metallography in Figure 5 and clearly indicates the phenomenon of carbon diffusing. Even though the temperature of the SCHAZ is lower than the initial phase transformation temperature, i.e., A_C1_ (665 °C), the increasing temperature led to the C diffusion, which resulted in the tempering and decomposition of martensite. As shown in Figure 10a,b, the SCHAZ and the BM have a similar phase structure, which is polygonal ferrite + island martensite, but the tempered martensite in the SCHAZ caused by C diffusion implies a lower hardness. In addition, the SCHAZ has a large temperature range, and thus a wide zone with temperatures between ~200 °C and 665 °C can be recognised as the SCHAZ, which mainly included the tempered martensite [24]. As a result, the softening behaviour can occur in a wide range (more than 2 mm), as shown in Figure 6.

The FEM simulation can directly show the temperature field of the sample after the welding cycle, which is difficult to achieve in Gleeble simulations. With the combination of calculated phase diagrams, the different zones can be recognised by the FEM simulation precisely according to A_C1_ and A_C3_. However, the error between the two simulation methods still exists. As shown in Figure 8b, the range of phase transition zones (higher than Ac_1_ temperature) was underrated during FEM simulations, which led to narrower CGHAZ, FGHAZ and ICHAZ values (between A_C1_ and A_C3_ temperatures) in the FEM results. The width of the ICHAZ after Gleeble simulation was about 1579.3 μm, but it was only 1125.0 μm after the FEM simulation. The difference between physical and numerical simulations can be attributed to the different heat sources and heat inputs in both FEM simulation and Gleeble simulation. Even though both simulation methods adopt resistance heating, the temperature controlling strategies are different. In the Gleeble simulation, the welding temperature was controlled by the thermal couples which were at 10 mm from the contact surfaces. The set temperature of the thermal couple was up to 1150 °C according to the thermal cycle from real welding. However, the FEM simulation needs to adjust the temperature of the burnt elements during the flashing process to ensure that these elements can be melted. Therefore, the heating range of the FEM simulation was narrower than the Gleeble simulation, which led to errors in the ranges of the simulated CGHAZ, FGHAZ and ICHAZ. Compared with the Gleeble simulation, the results of the FEM simulation are more theoretical.

However, both the numerical method and the physical method show satisfactory compatibility in the FBW simulation. Different zones can be clearly recognised by the combination of the calculated phase diagram and axial temperature distribution by the FEM, and thus expensive experiments can be avoided. Furthermore, the microstructures and mechanical properties of the HAZs can be studied through the physical simulation, which helps to adjust the welding process for real production. Meanwhile, the combined results of the calculated phase diagram and FEM simulation are beneficial to understand the difference in the HAZs in the Gleeble simulation. The combination of the two methods can be relied on to optimise process parameters, shorten the research period and reduce costs for real production experiments.

The FEM simulation revealed and solved the problem that the heating range of the Gleeble simulation is too large. Furthermore, the unstable thermal curve in Figure 2b does not appear in the FEM simulation. However, the undefined electrical conductivity and thermal conductivity at high temperature still seriously influence the result of the FEM simulation, which is not influential in the Gleeble simulation. Thus, it is concluded that the combination of these two simulations is more reliable than using only one.

## 4. Discussions

In the Gleeble simulation, the welding temperature was controlled by the thermal couples which were 10 mm from the contact surfaces. The set temperature of the thermal couple reached up to 1150 °C according to the thermal cycle from real welding. However, the FEM simulation only needs to adjust temperature of the burnt elements during the flashing process to ensure that these elements can be melted. Therefore, the heating range of the FEM simulation was narrower than the Gleeble simulation, which led to the errors in the ranges of the simulated CGHAZ, FGHAZ and ICHAZ.

During the Gleeble simulation, an isothermal process should be carried out to ensure the temperature of the joint; however, this process is not adopted in the FEM simulation. Furthermore, the thermal conductivity of the material will be different with the increased temperature, and this phenomenon is not reflected in the FEM simulation. Hence, the range of different HAZs given by the FEM and Gleeble simulations might be different.

In summary, the microstructure and mechanical properties of the DP590 wheel steel were discussed with a combination of physical and numerical simulations. In terms of the DP590 HAZs, four typical zones were clarified based on peak temperatures of welding cycles. The microhardness of the CGHAZ and FGHAZ was higher than the other zones because martensite transformation occurred in these two zones. The hardness of the ICHAZ was lower than the CGHAZ and FGHAZ, but it was still higher than the BM due to the combined action of residual martensite and refinement strengthening. However, the hardness of the SCHAZ was lower than the BM because of the tempered martensite, whose carbon diffusion occurs in tempering. The softening behaviours which occur (hardness decreases and width increases) in the welding of DP steels cannot be avoided. However, it can be controlled to a lower extent through the control of phase and grain transformation by the adjustment of welding thermal cycles, and both physical and numerical simulations can be referred to in order to optimise the welding parameters due to their own features and advantages.

However, some important issues need to be discussed in the future, such as residual stress. Residual stress always appears after welding. In general, residual stress might be different in ferrite and martensite zones [25]. In FBW-ed DP590 steel, the residual distribution in the HAZ will be not uniform because of the complex phases. Furthermore, the plastic deformation from the upsetting process directly causes residual stress. Therefore, the study of residual stress of flash butt welding of DP590 steel will be conducted in the future.

## 5. Conclusions

Combinational simulations of physical–numerical methodologies were carried out for flash butt welding of innovative DP590 wheel steel, and the two methods were highly complementary. The simulated results are as follows:

(1) The DP590 processed by the flash butt welding mainly had four types of HAZs. The CGAHZ and FGHAZ mainly contain lamellar martensite structures. The ICHAZ has fine ferrite grains and tempered martensite structures due to incomplete phase transition. The SCHAZ mainly has ferrite grains and island martensite structures, which is similar to the BM.
(2) The CGHAZ has the highest hardness due to its lamellar martensite structures and large prior austenite grains. The lamellar martensite and fine grains provide a high hardness (higher than the BM) for the FGHAZ and ICHAZ, respectively. The softening behaviour appeared in the SCHAZ, whose hardness decreased by 9.2% compared with the BM. The ultimate tensile strength of the FBW-ed sample decreased by 9.1%.
(3) The high heat input resulted in the diffusion of carbon elements during the flash butt welding process, and the carbon diffusion led to the tempering and decomposition of martensite islands in the SCHAZ. The microstructure and grain size of the SCHAZ is similar to the BM, i.e., ferrite + martensite, but the tempering and decomposition of martensite islands led to softening behaviour.

Even though the slight decrease in hardness of the SCHAZ may be acceptable for the end users of the wheel rim DP590, this softening phenomenon should be avoided as much as possible through optimised welding parameters. Here, the understanding of softening mechanisms helped us to modify and optimise the welding and hot-working process of this innovative DP590 steel. This study provides insights into welding high-strength dual-phase steel by high heat input and flash butt welding. Meanwhile, it offers a guideline for the combination of physical and numerical simulations of this welding method.

## Figures and Tables

**Table 1:** Chemical composition of DP590 wheel steel.

Elements	C	Mn	Cr	Si	Al	Ti	Nb	P	N	S	Fe
Wt. %	0.056	1.199	0.268	0.085	0.039	0.02	0.013	0.015	0.004	0.001	Bal.

**Table 3:** Load and boundary conditions.

Electrode Voltage (V)	Contact Interaction Potential (V)	Step Time (s)	Initial Temperature (°C)
220	0	1.2	20

## Data Availability

The data reported in this study can be provided on reasonable requests. Please address to the corresponding authors: L.Z. (lz131@uowmail.edu.au), C.M. (macheng01@hbisco.com), and J.H. (jianh@uow.edu.au).

## Supplementary Materials

The following are available online at https://www.mdpi.com/article/10.3390/ma16093513/s1. Figure S1. Gleeble data outputs (temperature-time curves) for Test No.2 and Test No. 3. Figure S2. Indication of different HAZs and hardness test zone. Figure S3. Calculated phase diagram of DP590 steel. Figure S4. Calculated temperature distribution of the sample. Table S1. Thermoelectric property and thermal conductivity.
