# Supplementary material for: Physical Simulation and Numerical Simulation of Flash Butt Welding for Innovative Dual Phase Steel DP590: A Comparative Study
Source: Materials (Basel). 2023 May 3;16(9):3513. doi: 10.3390/ma16093513 (PMC10180431; doi:10.3390/ma16093513)
Supplement: Supplementary file 1 [file materials-16-03513-s001.zip › materials-2355107-supplementary.pdf]

materials

MDPI

Supplementary Material

Physical Simulation and Numerical Simulation of Flash Butt Welding for Innovative Dual Phase Steel DP590: A Comparative Study

Jingwen Song [1], Lisong Zhu [2,*], Jun Wang [3], Yao Lu [3], Cheng Ma [4,*], Jian Han [2,*] and Zhengyi Jiang [2]

1 School of Materials Science and Engineering, Tianjin University of Technology, Tianjin 300384, China
2 School of Mechanical, Materials, Mechatronic and Biomedical Engineering, University of Wollongong, Wollongong, NSW 2522, Australia
3 Welding and Additive Manufacturing Centre, Cranfield University, Cranfield, MK43 0AL, UK
4 Technology Research Institute, HBIS Group, Shijiazhuang 050023, China
* Correspondence: lz131@uowmail.edu.au (L.Z.); macheng01@hbisco.com (C.M.); jianh@uow.edu.au (J.H.)

This work was financially supported by University of Wollongong & HBIS Group collaborative project (No. IRIS-200506001).

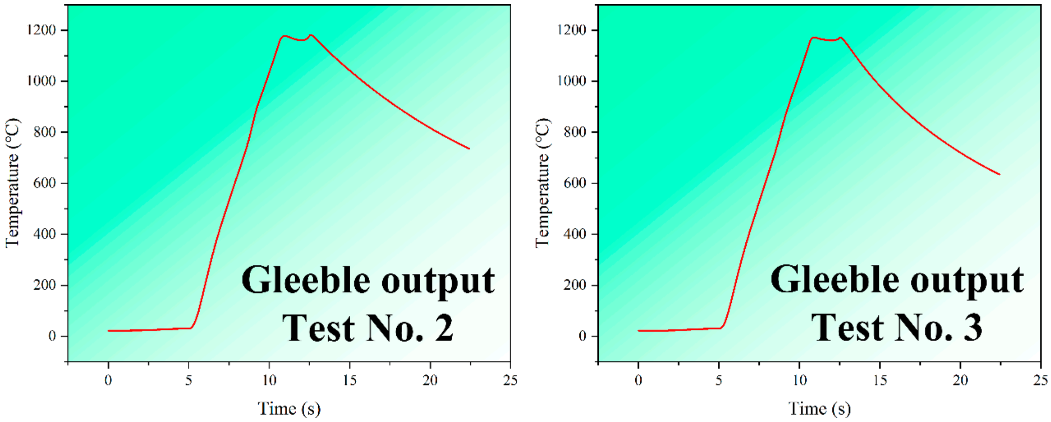

Figure S1. Gleeble data outputs (temperature-time curves) for Test No.2 and Test No. 3.

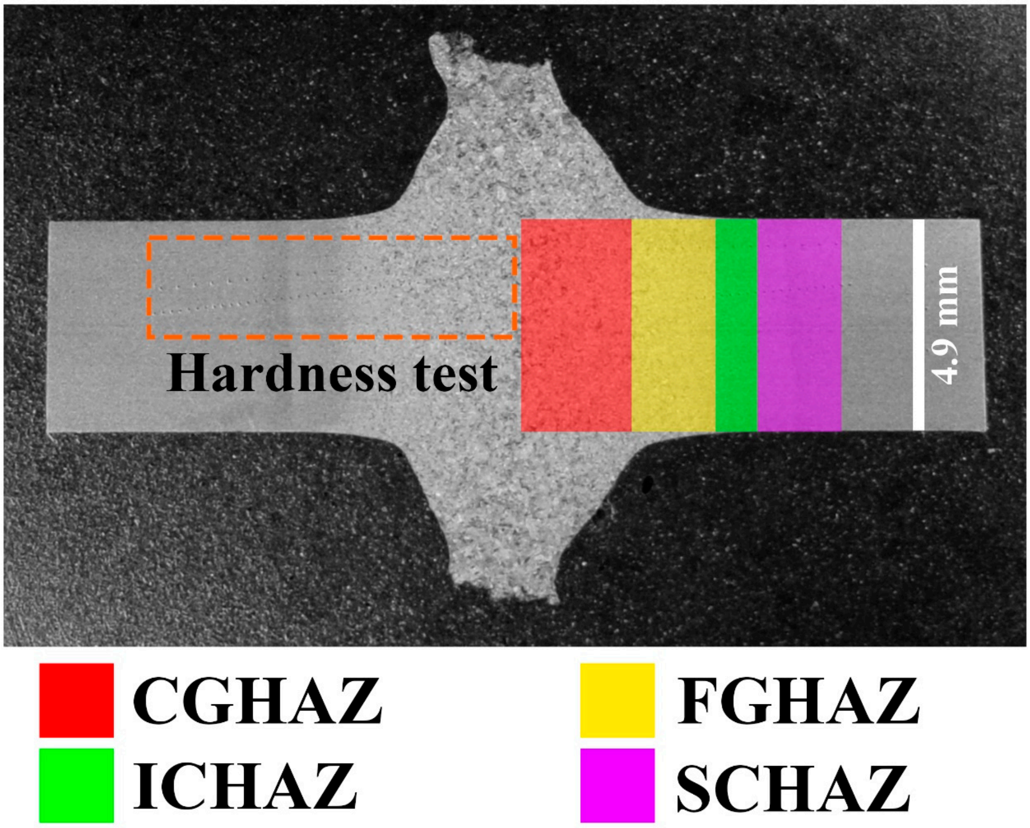

Figure S2. Indication of different HAZs and hardness test zone.

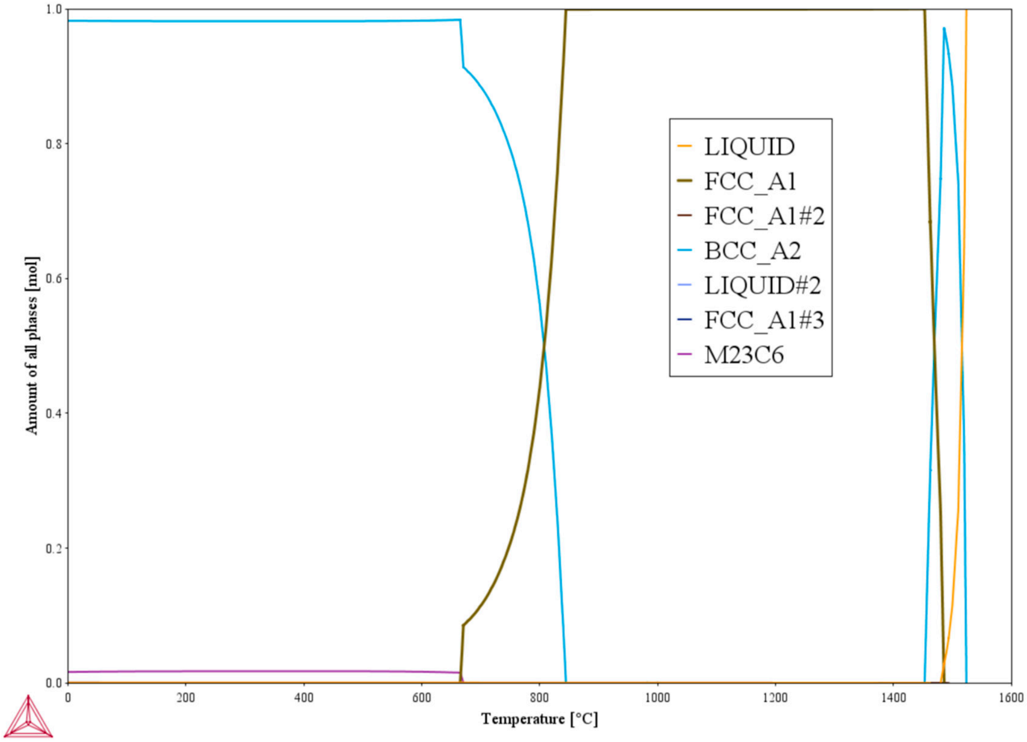

Figure S3. Calculated phase diagram of DP590 steel.

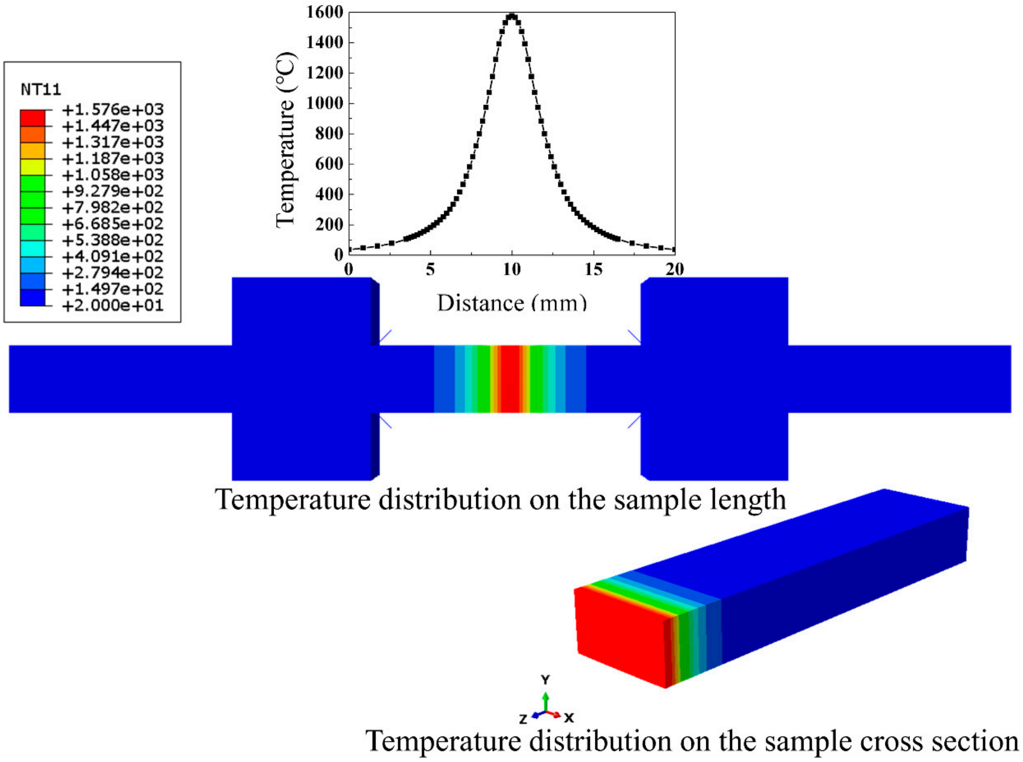

Figure S4. Calculated temperature distribution of the sample.

Table S1. Thermoelectric property and thermal conductivity.

Temperature (°C)	Thermoelectric property $(\Omega\cdot m)^{-1}\times10^{9}$	Thermal conductivity $(W/(m\cdot°C)\times10^{5})$
21	1.75	560
93	1.8	620
204	1.94	640
315	1.96	680
426	2.16	720
538	2.32	910
650	3.19	1300
760	7.35	3900
870	8.57	4800
982	10	5000
1093	12.2	5000
